# Mechanochemical Preparation of Magnetically Separable Fe and Cu-Based Bimetallic Nanocatalysts for Vanillin Production

**DOI:** 10.3390/nano11041050

**Published:** 2021-04-20

**Authors:** Paulette Gómez-López, Claudia Espro, Daily Rodríguez-Padrón, Alina M. Balu, Francisco Ivars-Barceló, Olvido Irrazábal Moreda, Clemente G. Alvarado-Beltrán, Rafael Luque

**Affiliations:** 1 Grupo FQM-383, Departamento de Química Orgánica, Campus de Rabanales, Universidad de Cordoba, Ctra Nnal IV-A, Km 396, 14014 Cordoba, Spain; gomezl.paulette@gmail.com (P.G.-L.); dailydggs@gmail.com (D.R.-P.); qo2balua@uco.es (A.M.B.); 2 Dipartimento di Ingegneria, Università di Messina, 98166 Messina, Italy; espro@unime.it; 3 Departamento de Química Inorgánica y Química Técnica, Facultad de Ciencias, UNED, Paseo Senda del Rey, 9, 28040 Madrid, Spain; accfib@hotmail.com (F.I.-B.); olvidoirrazabal@gmail.com (O.I.M.); 4 Facultad de Ingeniería Mochis, Universidad Autónoma de Sinaloa, Fuente de Poseidón y Prol. Angel Flores, S.N., Los Mochis, Sinaloa 81223, Mexico

**Keywords:** mechanochemical synthesis, mechanochemical extraction, green chemistry, solvent-free process, Cu nanoparticles, heterogeneous catalysis

## Abstract

A highly sustainable method for the preparation of supported iron oxide and copper nanoparticles (NPs) on a biomass-derived carbon by solvent-free mechanochemical process is reported. In-situ mechanochemically obtained extracts from orange peel could behave as a green reducing agent, allowing the formation of Cu metal nanoparticles as well as generating a magnetic phase (magnetite) in the systems via partial Fe^3+^ reduction. At the same time, orange peel residues also served as template and carbon source, adding oxygen functionalities, which were found to benefit the catalytic performance of mechanochemically synthesized nanomaterials. The series of magnetic Cu-Fe@OP were tested in the oxidation of trans-ferulic acid towards vanillin, remarkably revealing a maximum vanillin yield of 82% for the sample treated at 200 °C.

## 1. Introduction

Synthetic vanillin, currently produced from petro-based intermediates (glyoxylic acid and guaiacol), possesses a remarkable value as flavoring agent in cosmetic, pharmaceutical, food, and fine chemical industries. Ref. [1] Looking forward to minimize chemical waste and avoid non-sustainable synthetic methodologies, the scientific community has started to move towards the use of biomass residues as feedstock for vanillin production, [2,3] through catalyzed oxidation strategies employing green oxidants such as H_2_O_2_ or molecular oxygen. Several preeminent molecules derived from lignin including eugenol, isoeugenol, and trans-ferulic acid have been employed for the synthesis of vanillin via oxidation pathways. Specially, trans-ferulic acid is a non-toxic phenolic compound, highly abundant in lignocellulosic biomass, thus being a suitable candidate as feedstock for vanillin production [4].

Heterogeneous catalytic strategies employing nanomaterials, such as metal and metal oxides, have been explored for the transformation of trans-ferulic acid into vanillin [5]. In this regard, Cu based nanoparticles (NPs) have been widely investigated due to their chemical and physical properties, besides the earth-abundant and inexpensive characteristics of this metal [6]. Moreover, Cu nanoparticles supported on carbon have demonstrated to be a promising option, favoring the formation of highly dispersed NPs and resulting in synergistic interactions which could further boost the catalytic response [7]. In addition, the design of bimetallic nanocatalysts, by adding a second metal, could lead to tunable electronic and/or structural properties, hence modifying the catalytic performance, in terms of activity, selectivity, and stability [8]. Such types of catalytic systems have been widely investigated for biomass conversion reactions [9]. In particular, Fe–Cu bimetallic systems have demonstrated to be very promising options for their application in several processes, including higher alcohols synthesis [10,11,12]. Remarkably, the concomitant presence of iron oxide, in maghemite or magnetite form, could also provide magnetic features to the catalytic material, which certainly facilitate further recovery and reuse.

Recently, research endeavors have been focused on the preparation of more efficient catalysts and nanomaterials, paying special attention to the environmental impact of the synthetic procedure [13,14,15]. In particular, green synthetic approaches, employing natural extracts from plants, have exhibited outstanding results for the preparation of metallic nanoparticles including Au, Ag, and Cu [16]. Such plant extracts could play a crucial role as reducing agents and stabilizers, due to the presence of phyto-constituents, in particular antioxidants such as ascorbic acid, tocopherols, polyphenolic compounds, and terpenoids [17]. For instance, orange peel extracts have been employed as reducing agents to synthetize metallic Ag NPs [18].

Orange peel residues have been widely investigated due to their potential application to obtain value-added products (D-limonene, pectin) [19], biogas, [20] biofuel, [21] bio-absorbents [22], and active carbon [23]. Furthermore, orange peel wastes have been employed to obtain metal oxides nanoparticles, through sacrificial template approaches [24], and for the preparation of porous bio-sorbents [22].

Outstandingly, mechanochemical-assisted extraction methodologies could lead to highly efficient and environmentally friendly processes, reducing or even avoiding the use of solvents and additional reagents. In addition, it is also worth noting that mechanochemistry is a highly reproducible, clean, versatile, and simple approach. To date, mechanochemical methods for extraction of valuable chemicals have been reported for various agricultural residues, such as *Laurus nobilis* L. leaves [25], *Stephania tetrandra* S. Moore wastes [26], and *Platycodon grandiflorum* [27]. Mechanochemical extraction from agricultural wastes and in-situ formation of metallic and well dispersed nanoparticles, where the extracts could participate as reducing agent and the agricultural residue as carbon source and template, certainly represent a step further for the sustainable preparation of nanomaterials [14,28]. 

Considering the above-mentioned premises, herein we report a one-pot synthesis of carbon-supported bimetallic Cu and Fe NPs, via in-situ mechanochemical extraction from orange peel wastes. The herein proposed mechanochemical methodology represents one of the most sustainable alternatives, to the best of our knowledge, resulting in carbon-decorated Cu and Fe nanocatalysts. Such an approach is a simple, ecofriendly, clean, and non-toxic process, especially to obtain reduced Cu based compounds. As-obtained materials have been tested in the transformation of trans-ferulic acid into vanillin under mild conditions, using H_2_O_2_ as a green oxidant.

## 2. Materials and Methods

### 2.1. Chemical Reagents

Raw orange peels were obtained from the market, Cu(NO_3_)_2_ 3H_2_O (99.5% purity) and Fe(NO_3_)_3_·9H_2_O (99.5% purity) were acquired from Merk (Spain), trans-ferulic acid (99% purity) and hydrogen peroxide solution (50 wt.% in water) were purchased from Sigma-Aldrich (Spain). All the chemicals were used without any further purification steps.

### 2.2. Green Synthesis of Orange Peel-Derived Catalysts (Cu-Fe@OP)

Fresh orange peel, previously washed, was cut in small pieces of approximately 0.5 cm, and milled with 10% w.t. of Cu(NO_3_)_2_ 3H_2_O and 5% w.t. Fe(NO_3_)_3_·9H_2_O in the Emax ball mill model (Retsch), during 30 min at 900 rpm employing 10 iron balls (1 cm diameter). Subsequently, the samples were thermally treated in a calcination oven at different temperatures (100, 200, 300, and 400 °C), during 1 h under N_2_ atmosphere.

### 2.3. Catalytic Experiments

Cu-Fe@OP catalysts were tested in the oxidation of trans-ferulic acid to obtain vanillin. The experiments were performed at 90 °C, using 1.2 mL of H_2_O_2_ (50 wt.% in water) as green oxidant, 5 mmol of trans-ferulic acid, 8 mL of acetonitrile, and 0.1 g of the catalytic material. Complete conversion was achieved for all samples including blank runs since ferulic acid was found to decompose (i.e., decarboxylation) under the investigated conditions. Significant differences in selectivity were found for catalytic materials as compared to blank runs which provided negligible selectivity to vanillin.

### 2.4. Characterization

The structural characterization of the samples was accomplished, employing a Bruker D8 Discover X-ray diffractometer (Billerica, MA, USA) with Cu Ka radiation. The textural studies were conducted using the Porosimeter Micrometrics ASAP 2000 instrument (Haan/Duesseldorf, Germany). The morphology of the samples was evaluated employing by scanning electronic microscopy (SEM) in the JEOL-SEM JSM-7800 LV scanning microscope (Oberkochen, Germany). In addition, transmission electronic microscopy (TEM) images were recorded in a JOEL JEM 1400 instrument, assembled with a charge-coupled camera device (Oberkochen, Germany).

X-ray photoelectron spectra (XPS) were collected at room temperature using a Mg Kα (hν = 1253.6 eV) X-ray radiation source operated at 75 W (12.5 keV and 6 A), and a 7 channeltrons electrostatic hemispherical analyzer, from Scienta Omicron, refurbished by SPECS with a Phoibos 100 R4 analyzer technology (Berlin, Germany). The default detector voltage was kept constant at 2000 eV for all measurements and the spectra were acquired with pass energy of 20 eV and an energy step of 0.1 eV. The base pressure in the analysis chamber was kept below 5 × 10^−9^ mbar during the analyses. The binding energy scale was calibrated according to the adventitious C(1 s) peak at 284.6 eV for all the spectra.

Asymmetric Lorentzian, Gaussian–Lorentzian (60:40), and Gaussian–Lorentzian (20:80) line-shape backgrounds were used for the curve fittings of Fe2p, Fe3p, and Cu2p core levels, respectively. In all cases, spectrometer transmission function, cross section, and inelastic mean free path values from CasaXPS software were employed for quantitative calculations.

The analysis of the samples, taken from the catalytic experiments, was performed in a gas chromatograph Agilent Technologies 7890 A (Waltham, MA, USA) using a Petrocol TMDH column and a flame ionization detector (FID). Furthermore, identification of by-products was conducted using an Agilent 7820 GC/5977B GC/MSD (Waltham, MA, USA) system.

## 3. Results and Discussion

**Figure 1:**
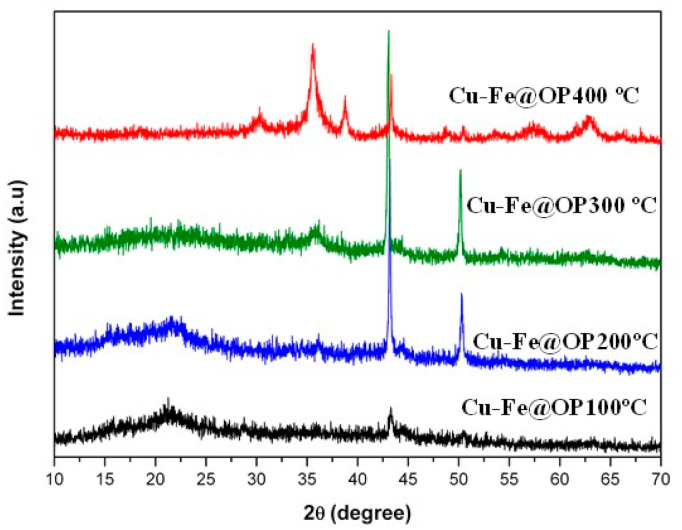
X-ray diffraction (XRD) patterns of Cu-Fe@OP100 °C, Cu-Fe@OP200 °C, Cu-Fe@OP300 °C, and Cu-Fe@OP400 °C samples.

A novel and sustainable protocol has been designed for the preparation of bimetallic supported nanoparticles, by employing orange peel as template and carbon source, while the in-situ mechanochemically obtained extracts favor the formation of metallic Cu [18]. X-ray diffraction (XRD) patterns of the prepared samples revealed the presence of two main peaks, located at 43.3° and 50.4°, related to (111) and (200) crystallographic planes of metallic Cu with cubic phase (PDF 04-0836, space group 225/Fm-3m), respectively. Such signals were found in all prepared materials, as shown in Figure 1. A broad band around 20.0°, which was observed for samples treated at 100 °C and 200 °C, could be attributed to the presence of amorphous carbon [7,19,21]. In turn, the aforementioned signal clearly decreased at higher temperature due to the partial elimination of the carbonaceous matrix.

In addition, the diffraction peaks related to metallic Cu displayed an intensity loss in the case of the sample calcined at 400 °C. Such results could be associated with the formation of CuO with monoclinic phase (PDF 48-1548 space group 15/C2-C) [7], as confirmed with the appearance of two main signals around 35.5° and 38.7° corresponding to (11-1) and (111) crystallographic planes. The formation of Cu oxidized species, despite the employed inert atmosphere, could be most likely related to the presence of oxygen functionalities, which are still present in the surface of the orange peel derived carbon (around 30% at 400 °C) [21].

**Figure 2:**
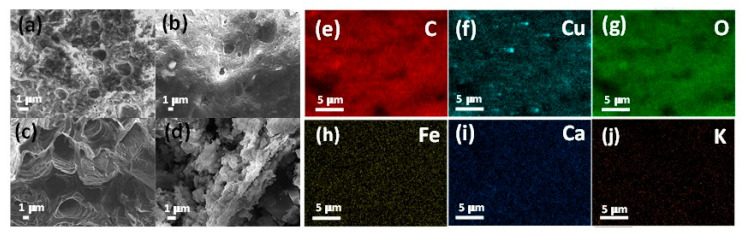
Scanning electron microscopy (SEM) micrographs of (**a**) Cu-Fe@OP100 °C, (**b**) Cu-Fe@OP200 °C, (**c**) Cu-Fe@OP300 °C, and (**d**) Cu-Fe@OP400 °C samples. SEM-mapping analysis of Cu-Fe@OP200 °C sample for (**e**) carbon, (**f**) copper, (**g**) oxygen, (**h**) iron, (**i**) calcium, and (**j**) potassium.

The morphology of the obtained materials was subsequently investigated by SEM and TEM analyses. As shown in Figure 2a–c, SEM micrographs of the Cu-Fe@OP catalysts displayed a hollow-type structure. Ref. [22] However, at higher temperature, namely at 400 °C, such structure collapsed (see Figure 2d), most likely due to cellulose and hemicellulose degradation between 250–360 °C. [29]

SEM-mapping analysis of Cu-Fe@OP200 °C sample was also carried out in order to investigate the elemental composition on the surface of the sample, as shown in Figure 2e–j. Such analysis revealed the presence of C, Cu, and O as most abundant elements in the materials, besides the concomitant appearance of Fe, Ca, and K. All elements were found to be homogeneously distributed on the catalyst surface, however, in the case of Cu, some agglomerated regions were detected, as clearly visualized in Figure 2f. The prominent presence of oxygen, despite the employed inert atmosphere for the synthetic approach, could be most likely related to the high oxygen functionalities in the carbon source at the investigated temperature (200 °C).

**Figure 3:**
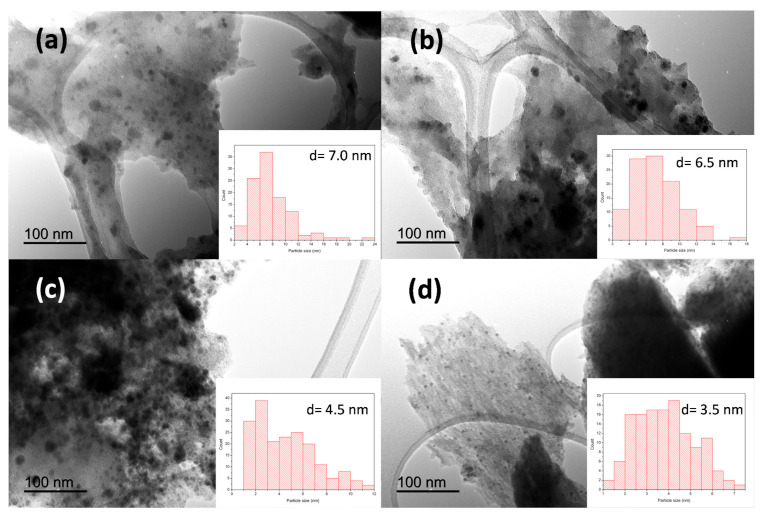
Transmission electron microscopy (TEM) images of (**a**) Cu-Fe@OP100 °C, (**b**) Cu-Fe@OP200 °C, (**c**) Cu-Fe@OP300 °C, and (**d**) Cu-Fe@OP400 °C samples. Inset shows the particles size histogram and the average particles size values.

TEM analysis of the samples revealed the formation of well-dispersed spherical nanoparticles embedded in a carbon matrix (Figure 3). The nanoparticles exhibited a main diameter lower than 10.0 nm for all the samples, as can be observed in TEM micrographs and in the inset histograms (Figure 3a–d). Interestingly, the temperature of the thermal treatment had a noticeable influence on the particle size distribution, going from a wide distribution for the sample treated at 100 °C, to a narrow distribution with a smaller particle size (3.5 nm) for the material calcined at 400 °C (See Figure 3d).

The textural properties of the samples were investigated by N_2_-physisorption analysis, as shown in Table 1. Remarkably, an increase in thermal-treatment temperature, from 100 °C to 400 °C, had a clear influence on the surface area, rising from 2 m^2^/g to 29 m^2^/g, for the Cu-Fe@OP100 °C and Cu-Fe@OP400 °C materials, respectively. Such trend could be associated with a higher porosity, promoted by cellulose and hemicellulose degradation [21]. Consequently, porous volume values also displayed a clear increment from 0.002 cm^3^/g to 0.070 cm^3^/g, for Cu-Fe@OP100 °C and Cu-Fe@OP400 °C, respectively, due to the partial elimination of organic moieties.

The chemical composition of representative samples, namely Cu-Fe@OP200 °C and Cu-Fe@OP300 °C, was obtained by quantitative analysis of XPS surveys acquired within the 1200–0 eV range, as shown in Table 2. Such study revealed the presence of carbon, nitrogen, oxygen, copper, and iron. It is worth noing the progressive increment in the Fe relative amount near surface, from 0.5 to 3.5%, by increasing the temperature of the thermal treatment, related to the loss of organic moieties from the material surface at higher temperature. In both catalysts, Cu content was found to be much lower than Fe concentration. Such result, together with XRD analysis, indicated that Fe nanoparticles were mainly deposited on the surface of the carbonaceous support, while Cu entities were most likely incorporated within the pores of the material (results from Cu-Fe@OP100 °C pointed to very low Cu content on the surface, not included).

Molar % were obtained from quantitative analysis of XPS surveys acquired at equivalent experimental conditions (see experimental section for details) from the following core levels: C1s, O1s, Cu2p, Fe2p, and N1s, integrated from the same spectrum for each sample (standard deviation < 0.2 eV).

**Figure 4:**
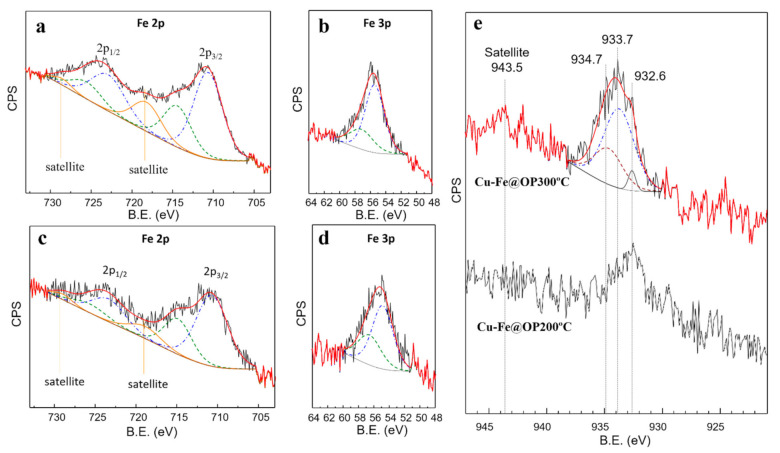
Curve fitting analysis of (**a**) XPS Fe2p and (**b**) Fe3p core levels of Cu-Fe@OP300 °C catalyst; curve fitting analysis of (**c**) XPS Fe2p and (**d**) Fe3p core levels of Cu-Fe@OP200 °C catalyst; (**e**) XPS Cu2p3/2 core level measurements for Cu-Fe@OP200 °C and Cu-Fe@OP300 °C samples.

The curve fitting of Fe2p and Fe3p core levels from XPS results of both Cu-Fe@OP200 °C and Cu-Fe@OP300 °C samples (Figure 4a–d) are consistent with the contribution of Fe^2+^ and Fe^3+^ species within the ranges of 68.5–73.2% and 26.8–31.5%, respectively. The analysis of the Fe2p region for the Cu-Fe@OP300 °C and Cu-Fe@OP200 °C catalysts showed the contribution of two different components for both Fe2p_1/2_ and Fe2p_3/2_ resolved signals, along with the characteristic satellite, at 5 eV and 8 eV above each core level maximum, respectively [30]. Focusing on the Fe2p_3/2_ signal, conventionally employed to determine the Fe^2+^/Fe^3+^ ratio [31,32,33], the two components appear at 710.67 eV (Fe^2+^) and 714.64 eV (Fe^3+^), with a standard deviation < 0.03 eV in both. The respective FWHM values, 4.09 eV and 4.38 eV (standard deviation < 0.2 eV), are characteristic of a broadening associated to unresolved multiplet splitting [34].

Fe^2+^/Fe^3+^ ratio obtained from the calculations upon the Fe2p region were confirmed by curve fitting analysis of the Fe3p core level for both samples, which due to the low spin-orbit coupling constant, consist of a single band including the unresolved Fe3p_1/2_ and Fe3p_3/2_ signals. Fixing the physical parameters (FWHM, asymmetry factor and Gaussian–Lorentzian ratio) according to previously reported calculations on the same region [29], two components with approximately 2 eV separation, at 55.4 (FWHM of 2.9) and 57.1 eV (FWHM of 3.6) binding energies were obtained, corresponding to Fe^2+^ and Fe^3+^ species, respectively. This likely corresponds to a magnetite (Fe_3_O_4_) phase present in the materials (not seen in XRD experiments due to the low iron content), in good agreement with the dark-black-color of the samples. Fe^3+^ species from the iron precursor were partially reduced by the orange peel extract to generate the observed Fe^2+^ species.

From XPS results obtained for Cu2p3/2 core level, the presence of Cu^2+^ was confirmed, as shown in Figure 4e for the Cu-Fe@OP300 °C catalyst, exhibiting the characteristic satellite peak at 942.0 eV [35]. A curve fitting analysis on the Cu2p3/2 region was performed for Cu-Fe@OP300 °C. Strictly applying the constrains for binding energy, FWHM, and line-shape reported for advanced analysis of Cu2p_3/2_ [36], the signal was consistent with three main components centered at 932.6, 933.7, and 934.7 eV, related to Cu reduced species, Cu^2+^ oxide and Cu^2+^ hydroxide species, respectively, according to the binding energy positions and the energy differential increments among them [37]. Thus, near surface contents of 4.1% (Cu reduced species), 66.7% (Cu^2+^ oxide), and 29.2% (Cu^2+^ hydroxide) were determined on Cu-Fe@OP300 °C. A meaningless Cu^+^ contribution (≤0.3%), with a characteristic component at 932.1 eV, could not be completely ruled out. Such results, together with XRD data, indicated that most of the metallic Cu is located within the pores of the material, while Cu on the material surface was found to be mainly as oxidized species. In addition, since the maximum for the Cu2p_3/2_ core level signals in Cu-Fe@OP200 °C is centered at the same binding energy of the Cu^0^ component found in Cu-Fe@OP300 °C (Figure 4e), the presence of Cu^0^ appears to be consistent, observed from the reduction of Cu^2+^ by the orange peel extract under mechanochemical conditions. Nevertheless, considering the Cu2p_3/2_ peak broad in those spectra, Cu^+^ species are likely to be coexisting. 

**Figure 5:**
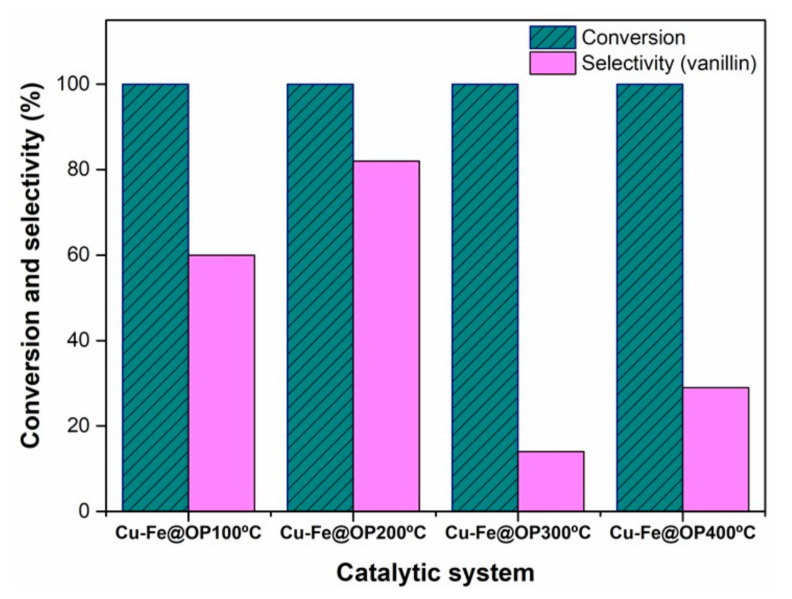
Catalytic performance of Cu-Fe@OP materials in the oxidation of trans-ferulic acid into vanillin.

**Figure 6:**
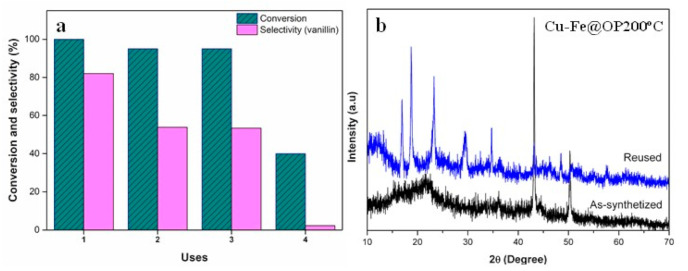
(**a**) Catalytic performance of Cu-Fe@OP200 °C material in the oxidation of trans-ferulic acid into vanillin over four reaction runs. (**b**) Post-characterization XRD analysis of the recovered Cu-Fe@OP200 °C catalyst, in comparison with the fresh sample.

The catalytic performance of the synthesized Cu-Fe@OP materials was explored in the oxidation of trans-ferulic acid into vanillin, using H_2_O_2_ as greener oxidant, under sustainable conditions based on a previously reported work from our research group [5]. Blank runs provided almost negligible selectivity to vanillin (<5%) under the investigated optimum reaction conditions, with other relevant products observed including vinyl guaiacol and lignin-like oligomers (dimers and trimmers). Comparably, materials resulted to be remarkably selective for the oxidation of trans-ferulic acid into vanillin (Figure 5 and Figure 6). Attending to the obtained selectivity values towards vanillin, the synthesized samples could be divided into two groups, namely the samples treated at lower temperature (Cu-Fe@OP100 °C, Cu-Fe@OP200 °C), and samples calcined at higher temperature (Cu-Fe@OP300 °C, Cu-Fe@OP400 °C). In particular, the higher selectivity observed for the samples treated at temperatures below 200 °C could be most likely associated to two main reasons: (1) higher amorphous content and (2) higher content of oxygen functionalities on the materials surface, for the samples treated at lower temperature. Indeed, various examples had demonstrated the improved catalytic efficiency of amorphous phases, as compared with their crystalline analogues, most likely due to higher flexibility of the structure, which could lead to a greater reactivity in oxidation process [38]. In addition, several studies had also described the effect of oxygen functionalities, as active sites, on the progress of catalyzed oxidation reactions, mimicking graphene oxide behavior [39,40]. In particular, for Cu-Fe@OP200 °C, selectivity values of 82% were reached. Remarkably, such value was found to be higher than the ones obtained following a fermentative strategy [40], and comparable to those ones employing Cu based materials, including MOP [41], MOF [42], and metal-oxides [5]. Furthermore, the increase in selectivity observed for Cu-Fe@OP400 °C, as compared to Cu-Fe@OP300 °C, could be most likely attributed to the presence of Cu oxidized species in the sample treated at 400 °C, together with a higher surface area, smaller particle size, and narrower particle size distribution observed for this material.

Additionally, reusability studies of Cu-Fe@OP200 °C were accomplished in order to investigate its stability, together with a post-characterization analysis (Figure 6). Outstandingly, the magnetic properties of the synthesized material greatly facilitate its recovery by simply using a magnetic field. Even if after the first use, the selectivity towards vanillin decreased from 82% to 53%, the sample retained an acceptable catalytic behavior with high conversion values of approximately 95% for the second and third cycle uses. However, after the fourth use, a clear deactivation of the sample was observed. XRD analysis of the recovered Cu-Fe@OP200 °C material was conducted, revealing the formation of Cu(OH)_2_ with orthorhombic crystalline structure (PDF 35-0505, space group 66/CmCm), corresponding to a spertinite phase (See Figure 6b). Such results could be interpreted in terms of low stability of Cu species under hydrogen peroxide environment, clarifying the reason for the catalyst deactivation after three runs. Comparatively, the iron oxide phase remains almost unaltered with a slightly higher contribution of Fe^3+^ (via oxidation of Fe^2+^) after the reaction with hydrogen peroxide, retaining in any case the magnetic features of the fresh catalyst. In view of the oxidation of Cu reduced species (mostly Cu^0^) to Cu(OH)_2_ during reaction, correlating well with a decrease in vanillin selectivity, such species seem to be the main catalytically active species responsible for the high vanillin selectivity in the systems under the investigated conditions.

## 4. Conclusions

A series of magnetically separable Cu-Fe@OP catalytic materials have been successfully synthesized using biomass orange peel wastes, as template and carbon source. Remarkably, the employed mechanochemical protocol resulted in the in-situ extraction from orange peel wastes, giving rise to an alternative green reducing agent, which further leads both to the formation of metallic Cu nanoparticles as well as to a magnetic phase (magnetite) from the partial reduction of the Fe (III) precursor. This methodology represents a highly sustainable approach, which could be translated to other biomass-derived wastes. The employed temperature in the thermal treatment of the samples, resulted to be a critical factor, affecting not just the surface area, crystal structure, and particle size, but also directly influencing the catalytic performance. In general terms, the catalytic activity of the samples resulted to be favored, when temperatures below 200 °C were employed during the synthetic step. Such results were interpreted considering the higher amorphous content and the greater presence of oxygen functionalities in the samples obtained at lower temperature. Noteworthy, this work could contribute to broadening current knowledge [43,44] on lignocellulosic biomass valorization strategies.

## Figures and Tables

**Table 1:** Textural properties of Cu-Fe@OP samples.

Catalyst	S_BET_^a^ (m^2^/g)	V_BJH_^b^ (cm^3^/g)
Cu-Fe@OP100 °C	<5	0.002
Cu-Fe@OP200 °C	<5	0.011
Cu-Fe@OP300 °C	7	0.026
Cu-Fe@OP400 °C	29	0.070

S_BET_^a^: specific surface area calculated by the Brunauer−Emmett−Teller (BET) equation. V_BJH_^b^: pore volumes calculated by the Barret−Joyner−Halenda (BJH) equation using the adsorption branch of the isotherm.

**Table 2:** Chemical compositions obtained by X-ray photoelectron spectra (XPS) analysis.

Sample	C (%)	O (%)	Cu (%)	Fe (%)	N (%)
Cu-Fe@OP200 °C	71.9	25.3	0.2	1.7	0.9
Cu-Fe@OP300 °C	68.0	26.0	0.7	3.5	1.8
